# Self-sufficient self-oscillating microsystem driven by low power at low Reynolds numbers

**DOI:** 10.1126/sciadv.abj0767

**Published:** 2021-10-27

**Authors:** Farzin Akbar, Boris Rivkin, Azaam Aziz, Christian Becker, Dmitriy D. Karnaushenko, Mariana Medina-Sánchez, Daniil Karnaushenko, Oliver G. Schmidt

**Affiliations:** 1 Institute for Integrative Nanosciences, Institute for Solid State and Materials Research Dresden, Leibniz IFW Dresden, 01069 Dresden, Germany.; 2 Material Systems for Nanoelectronics, Chemnitz University of Technology, 09107 Chemnitz, Germany.; 3 Research Center for Materials, Architectures and Integration of Nanomembranes (MAIN), TU Chemnitz, Rosenbergstraße 6, 09126 Chemnitz, Germany.; 4 Nanophysics, Faculty of Physics, TU Dresden, 01062 Dresden, Germany.

## Abstract

Oscillations at several hertz are a key feature of dynamic behavior of various biological entities, such as the pulsating heart, firing neurons, or the sperm-beating flagellum. Inspired by nature’s fundamental self-oscillations, we use electroactive polymer microactuators and three-dimensional microswitches to create a synthetic electromechanical parametric relaxation oscillator (EMPRO) that relies on the shape change of micropatterned polypyrrole and generates a rhythmic motion at biologically relevant stroke frequencies of up to ~95 Hz. We incorporate an Ag-Mg electrochemical battery into the EMPRO for autonomous operation in a nontoxic environment. Such a self-sufficient self-oscillating microsystem offers new opportunities for artificial life at low Reynolds numbers by, for instance, mimicking and replacing nature’s propulsion and pumping units.

## Teaser

Fast autonomous rhythmic motion is achieved in water for an artificial microsystem inspired by biologically relevant frequencies.

## INTRODUCTION

A few recent works have shown biomimetic, self-powered, and shape-transforming autonomous oscillating systems based on various physical principles. Self-oscillating walking gels made of stimuli-responsive hydrogels (HGs) and a chain of oscillating chemical reactions (Belousov-Zhabotinsky reaction) with oscillation frequencies up to 10 mHz have been demonstrated by Maeda *et al.* (*1*). The Octobot developed by Wehner *et al.* (*2*) has used microfluidic logic to oscillate at 50 mHz while opening and closing pneumatic actuators in a fully autonomous way. Likewise, McGovern *et al.* (*3*) demonstrated a self-oscillating autonomous system by integrating actuators and energy sources and driving circuitries into a single device. The 10-cm-long system was equipped with a conventional Zn-Mn battery cell, a conventional electronic circuit, and electroactive polypyrrole (PPy) actuators used as fins to propel the system in a water-filled tank. The embedded energy source provided the required energy for the Si-based electronics dictating the actuators to oscillate at a certain frequency. The power requirements (high operating voltages > 1.5 V and high operating currents > 1 mA) of conventional electrical circuits are incompatible with biological systems (*4*) as they cause water splitting or unwanted redox reactions. The other electromechanical oscillating bilayer systems are of Microelectromechanical systems (MEMS) (*5*) or thermal (*6*) switching type. These kinds of oscillating systems are either not compatible with liquid environment (hence cannot generate any flow), low amplitude, or slow.

In addition, there are more challenges to tackle at small scale. As the size decreases, viscous forces start to dominate over inertial forces, and a nonreciprocal undulatory movement of an object is necessary to create flow in the surrounding liquid environment (*7*). This further implies the importance of implementing a biocompatible self-sufficient system oscillating at several hertz and operating at low power in the low Reynolds number regime for biomimicry of small cells or organisms (*1*). Furthermore, the efficiency of chemical reactions for such soft actuating systems has to be enhanced, while the reagent toxicity and the overall system dimensions should be reduced.

Here, we create a self-oscillating autonomous soft PPy microactuator system that is driven by less than 1 V (versus Ag/AgCl), requires extremely low power (~10 μW or ~0.1 mcal each minute), and can produce electromechanical relaxation oscillations at biorelevant frequencies. Since PPy actuation is based on the diffusion of Na^+^ or other cations into and out of the polymeric matrix and hence expanding and shrinking the polymer, PPy actuators could function in various ion rich salt solutions such as cell culture medium, blood plasma, and urine (*8*). The few reports show that PPy self-oscillations exhibit low amplitudes of several millivolts and cannot produce mechanical motions. Furthermore, previous reports have demonstrated rather slow (around 0.024 Hz) electrochemical oscillations (*9*, *10*). The power consumption of our system compares to a running ant that consumes ~86 μW of metabolic power (~1 mcal each minute) (*11*) or a swimming calanoid copepod plankton that consumes about ~100 μW (~1.4 mcal each minute) (*12*). Furthermore, the system is powered either by an external power source or an embedded Ag-Mg battery (*13*). The latter provides autonomy and biocompatibility as Ag and Mg have previously been used safely in subcutaneous batteries and implants (*14*, *15*).

**Fig. 1.**
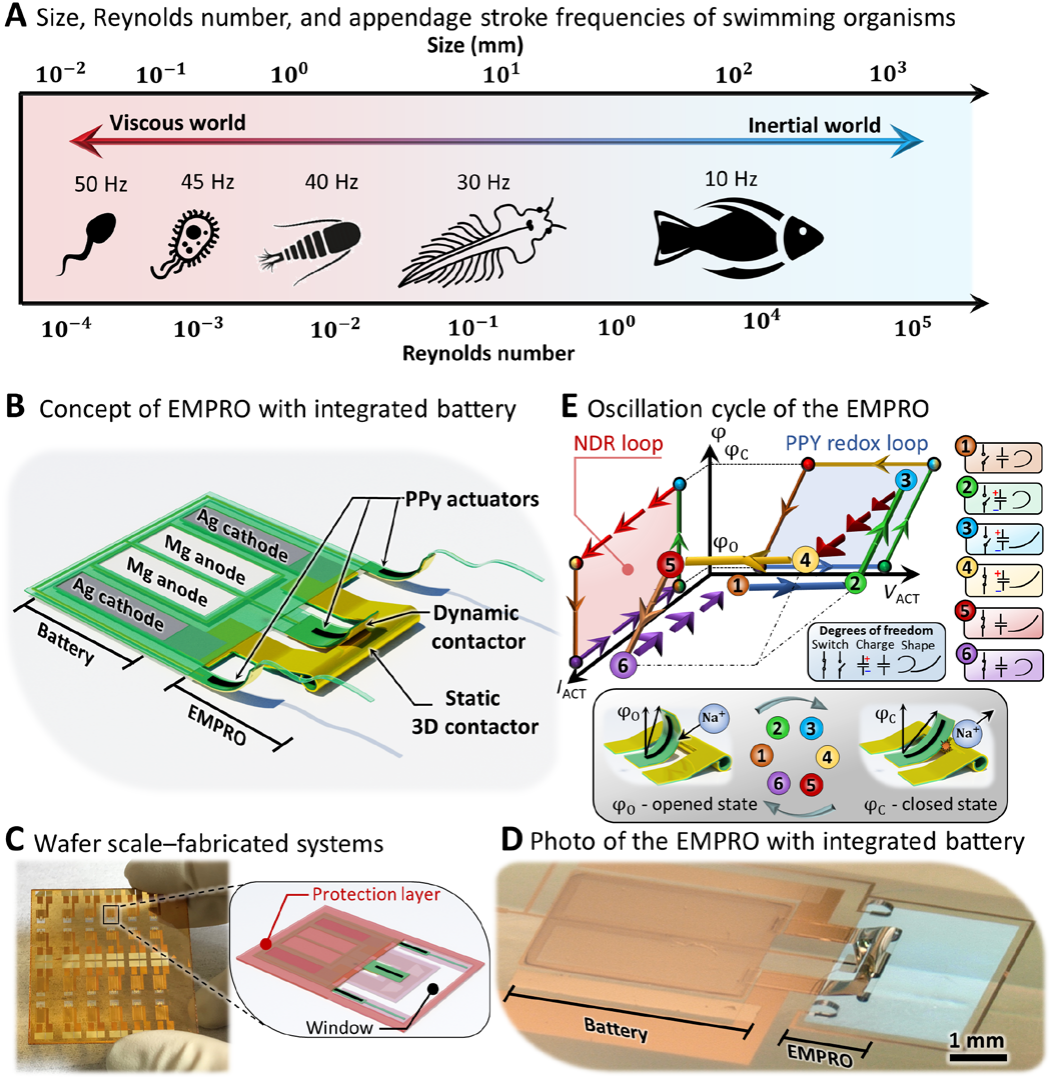
Swimming organisms and concept/realization of autonomous EMPROs. (**A**) Size versus Reynolds number of swimming organisms as the stroke frequency declines with increasing size, organisms from left to right: spermatozoon, paramecium, calanoid copepod, brine shrimp, and fish. (**B**) 3D sketch concept of the EMPRO with the battery. (**C**) EMPROs along with the energy sources and protecting photoresist layer fabricated on a 50 mm by 50 mm glass substrate before self-assembly (left) and the 3D sketch of a single device (right). (**D**) A photographic image of the EMPRO and the battery under protective photoresist after delamination and the self-assembly of the contactor. (**E**) Oscillation cycle of the EMPRO depicted on a 3D diagram of voltage across the EMPRO (*V*), current through the EMPRO (*I*), and deflection angle (φ). The EMPRO goes through six states for the oscillation. A constant current first electrically charges the PPy supercapacitor (1 → 2), then the PPy reduces and expands as voltage increases further (2 → 3), and upon further expansion, the switch closes, and a current surge occurs as the PPy supercapacitor begins to discharge (3 → 4); hence, the voltage across the PPy supercapacitor drops (4 → 5), as the voltage decreases further the PPy reaches oxidation voltage and contracts (5 → 6), and upon further contraction, the switch opens, the current drops to the initial value (6 → 1), and the cycle continues. Photo credit: Farzin Akbar, Institute for Integrative Nanosciences, Leibniz IFW Dresden.

Relaxation oscillations can be found ubiquitously in biological systems such as heart pulsations (*16*, *17*), muscle contractions (*18*), and neural oscillations (*19*). Here, the autonomous biomimicking relaxation oscillation is achieved through a specific set of design parameters that drives the integrated electromechanical parametric relaxation oscillator (EMPRO) into a nonequilibrium state. In this way, solely relying on a nontransistor-based architecture, all the available power is efficiently fed into the oscillation of the system with a rhythmic frequency comparable to its biological counterparts (Fig. 1A). Compared to EMPRO, conventional electrical and mechanical relaxation oscillators such as the neon-lamp vacuum tube oscillators (*20*), multivibrators (*21*), electric bells (*22*), or the series dynamo (*23*) require high operating voltages and currents. Furthermore, they are bulky, not suitable for integrated microsystems, and not biocompatible especially when working in fluids.

The EMPRO and the battery are combined and processed together on a single chip as shown in the concept sketch (Fig. 1B). The mechanical switch of the EMPRO is made of a tube-like static three-dimensional (3D) contactor that is placed directly under the dynamic contactor. The dynamic contactor is attached to an electrically responsive soft PPy polymeric microactuator (*8*, *24*, *25*) that is capable of reshaping at high speed (full actuation cycle under 200 ms) (*26*) while requiring low power for operation (ca. $400\;\frac{\mu W}{{\text{cm}}^{2}}$ ). The low power and the fast response of the PPy actuator are ideal for mimicking the oscillation of small-scale biological systems. The EMPROs are defined and patterned in a flat layout on a glass substrate by standard microfabrication and lithography techniques (Fig. 1C and fig. S1). The multilayer system comprises a shapeable polymer stack with sacrificial layers (SLs), swelling HGs, and soft polyimide (PI) and SU8-2000 reinforcing components. The PI and swelling HG polymer elastic moduli were reported to be 3.2 GPa and 4.5 MPa, respectively (*27*). For the SU8-2000, we assume an elastic modulus of 2 GPa (*28*). Although these elastic moduli are higher than that of biological tissues (~GPa compared to ~kPa), the low thickness of the used materials (less than 1 μm) ensures the softness and low bending rigidity of the EMPRO devices.

Upon selective removal of the SLs, the actuators and contactors are released from the substrate, and the built-in strain causes the EMPRO system to self-assemble into a refined 3D architecture (Fig. 1D, details explained in Materials and Methods).

The EMPRO oscillations undergo a six-state operation cycle in three degrees of freedom (switch state, capacitor charge, and actuator shape) (Fig. 1E). First, the Au/PPy as an embodied supercapacitor starts to charge electrically (1 → 2). At a certain voltage, PPy is electrochemically reduced (2 → 3), which leads to a flattening of the actuator as cations in the solution are driven into the polymer matrix. This causes a deflection of the dynamic contactor from φ_O_ toward the 3D static contactor. At the angle φ_C_, both contactors establish an electrical connection that leads to a rapid discharge current (3 → 4). The PPy supercapacitor begins to discharge electrically (4 → 5) until the onset potential for electrochemical oxidation of PPy is reached. Last, the PPy enters the oxidation region leading to the contraction of the actuator (5 → 6) as the cations in the polymer matrix diffuse back into the solution. As a result, the dynamic contactor deflects away from the static contactor, and the electrical connection is lost, leading to a rapid decrease of the electrical current (6 → 1) and the beginning of the next cycle.

**Fig. 2.**
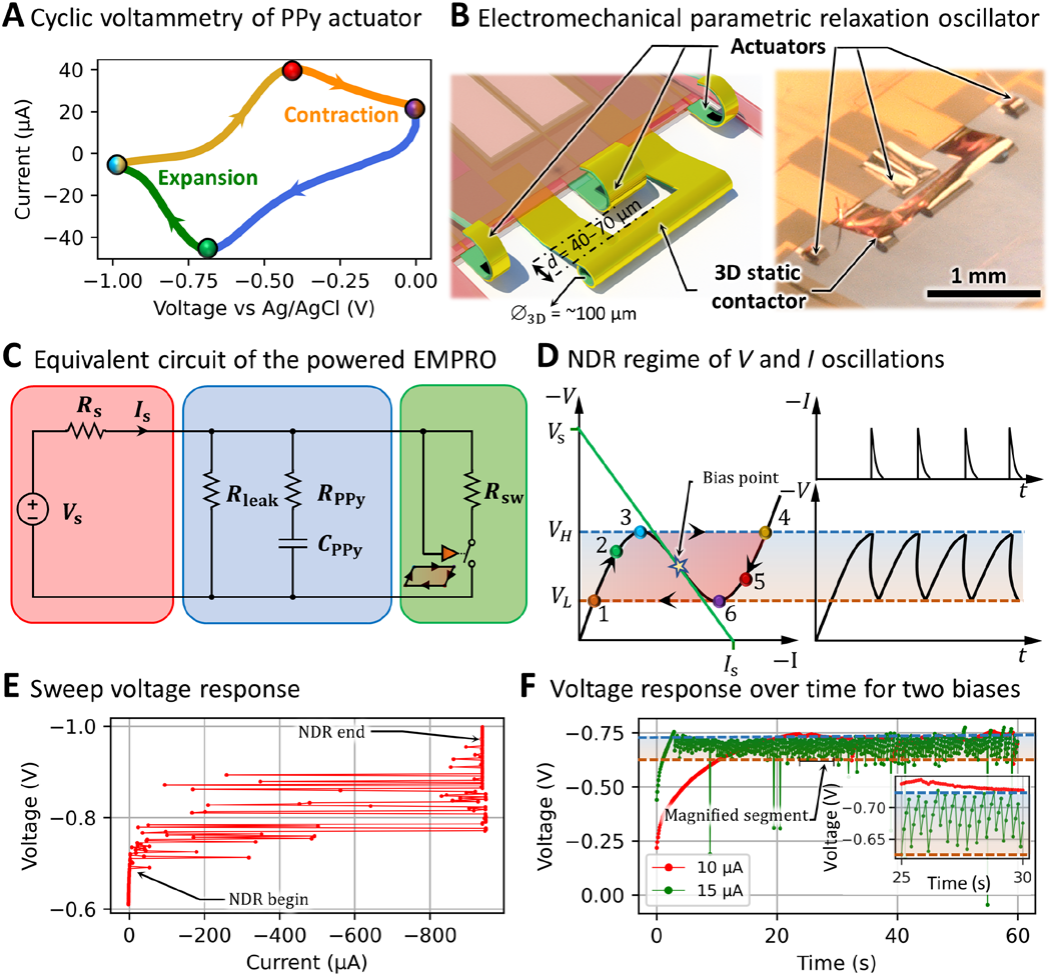
Theoretical and experimental results of EMPRO. (**A**) Oxidation-reduction diagram of PPy. Expansion and contraction occur in the green and orange regions, respectively, leading to redox hysteresis. (**B**) 3D self-assembled EMPRO concept and its realization. (**C**) EMPRO-equivalent electrical circuit powered by an energy source. (**D**) *IV* curve indicating the negative resistance and bias line of the EMPRO along with the response of *V* and *I* over time. (**E**) Voltage sweep from 0 to 1 V. Oscillations occur and stop as the bias enters and exits the NDR region. (**F**) EMPRO voltage response for two bias currents of 10 and 15 μA over a time span of 60 s. The inset is a 5-s segment of the EMPRO response showing oscillations of 2.5 Hz. Photo credit: Farzin Akbar, Institute for Integrative Nanosciences, Leibniz IFW Dresden.

This electromechanical oscillation is enabled by two hysteresis loops, namely, the PPy redox cycle and the negative differential resistance (NDR) loop. The NDR occurs when the two contactors touch and cause an electrical short in the system. Moreover, the PPy electrochemical supercapacitor (*29*) acts as the inertial element of the relaxation oscillator as reduction and oxidation only take place at the corresponding values of the electrochemical potentials (Fig. 2A).

## RESULTS

The low power and low operating voltage of the PPy actuators are directly evident from the cyclic voltammetry measurement (Fig. 2A). Most of the expansion occurs after the reduction peak below −0.7 V versus Ag/AgCl (green), and most of the contraction happens after the oxidation peak above −0.45 V versus Ag/AgCl (orange). This leads to the hysteresis of the voltage-deflection angle (Fig. 1E and fig. S2A) where it is observed that the EMPRO can have a deflection angle change of 50° during PPy electrochemical reduction. The EMPRO is a mechanical and an electrical oscillator; therefore, the generated oscillating signals can be applied to any other electromechanical components, such as extra actuators mimicking undulatory movement of flagella (artificial flagella) at the sides of the EMPRO (Fig. 2B).

An equivalent electrical circuit of the EMPRO driven by an energy source is illustrated in Fig. 2C. The relaxation oscillator is biased into the NDR region (Fig. 2D) by the energy source *V*_s_ through the resistance *R*_s_ providing a constant current *I*_s_. Since the actuation expansion of the PPy occurs mostly between −0.7 (reduction peak) and −1 V (versus Ag/AgCl), the negative resistance switching needs to occur in this interval as well [see Fig. 2 (A and D)]. Therefore, the critical system parameter, namely, the distance between the 3D static contactor and the dynamic contactor, was tuned between *d* = 40 and 70 μm where switching occurred at *V*_H_ = −0.72 and −0.79 V (versus Ag/AgCl), respectively, while the diameter of the 3D static contactor was optimal at Φ_3D_ = ~100 μm (Fig. 2B).

Since the EMPRO is a parametric relaxation oscillator, its geometry plays a major role in the occurrence of oscillations. Therefore, the geometric parameters were defined and tuned. For example, the shape of the static 3D contactor is achieved by tuning the thickness and shape of the swelling HG layer and the reinforcing PI layer. A thicker swelling HG layer would result in a tighter tube with a smaller diameter, whereas a thicker reinforcing PI layer leads to a larger tube with a bigger diameter (*30*). The thicknesses of these layers are tuned in the spin-coating step, before photopatterning. Furthermore, the distance between the dynamic and static contactor is tuned precisely in the lithography step so that the negative resistance occurs at the reduction point where most of the actuation takes place. The geometry of the PPy also plays an important role in the curvature of the actuators and the current consumption of the EMPRO. The current consumption increases with larger PPy areas. This would mean that dynamic contactors with larger PPy areas would need to be biased with more electrical currents to reach the oscillation angle φ_𝐶._ After obtaining the right geometrical parameters of the EMPRO system, we have fabricated an array of 24 devices, followed by the PPy deposition and self-assembly on a glass wafer of 50 mm by 50 mm, achieving a fabrication yield of 70% (fig. S3). The parameter tuning results in electromechanical oscillations around the operation voltage of approximately −0.7 V (versus Ag/AgCl reference electrode). The recurring charging and discharging of the PPy supercapacitor (*C*_PPy_) are triggered by the ON and OFF states of the switch. The corresponding time constants for charging and discharging are *t*_c_ = (*R*_s_ + *R*_PPy_)*C*_PPy_ and *t*_d_ = (*R*_sw_ + *R*_PPy_)*C*_PPy_, respectively. This leads to periodic current surges and output voltage oscillations between two values of *V*_L_ and *V*_H_ (Fig. 2D; *I* versus *t* and *V* versus *t* diagram). For the oscillations to occur, the EMPRO needs to be biased in the NDR region. The oscillation behavior of an EMPRO during a voltage ramp is shown in Fig. 2E. Gradually increasing the voltage from zero, oscillations start to occur when the EMPRO enters the NDR region (around −0.7 V versus Ag/AgCl). Once the bias voltage leaves the NDR region (around −0.9 versus Ag/AgCl), the oscillations stop. In a second trial, the EMPRO is biased with two constant currents of 10 and 15 μA. The constant current mode used to bias the EMPROs allows the PPy supercapacitor to be charged over time to reach the NDR region; however, if the current is too low, the parallel leakage resistance would prevent the bias in the NDR region. As shown in Fig. 2F, for *I*_s_ = 15 μA, the EMPRO is in the NDR region after being charged for a few seconds, causing voltage oscillations to occur at 2.5 Hz between −0.65 and −0.75 V (versus Ag/AgCl). For *I*_s_ = 10 μA, hardly any oscillations occur as the system is biased away from the NDR region. The behavior of the EMPRO at these current biases, along with *I*_s_ = 20 and 30 μA, can be found in movie S1. In this video, the electromechanical oscillations of the EMPRO are directly transferred to the mechanical oscillations of the artificial flagella integrated at two sides of the system.

**Fig. 3.**
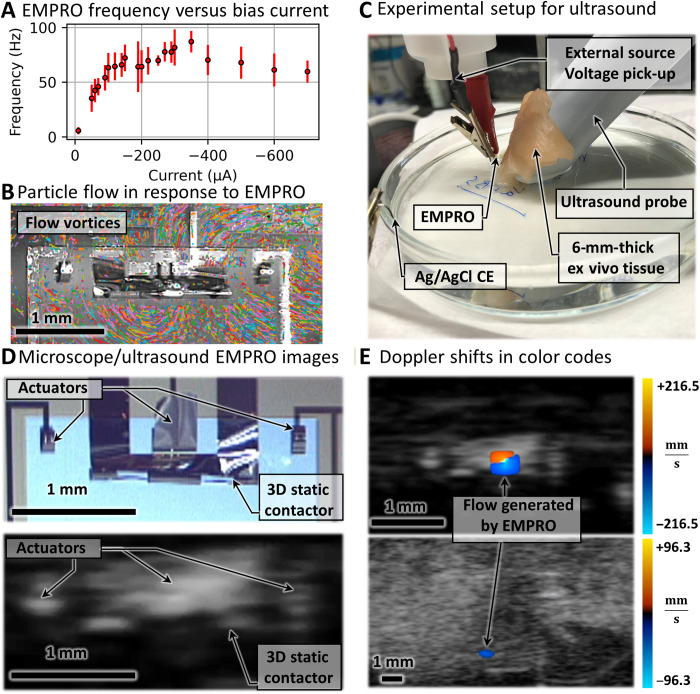
Flow generation by the EMPROs. (**A**) Average and SD of the frequency response versus bias currents (10 to 700 μA) of five EMPROs. (**B**) Vortex flow generated by oscillating EMPRO at 95 Hz. (**C**) Experimental setup for B-mode and Doppler-mode US experiments. (**D**) Stereoscopic microscope image of the EMPROs and the B-mode US recreation of it. (**E**) Flow generated by the EMPROs represented in color Doppler-mode without (top) and with (bottom) the ex vivo chicken tissue. Photo credit: Farzin Akbar, Institute for Integrative Nanosciences, Leibniz IFW Dresden.

The frequency of EMPRO oscillation is ultimately limited by the rate at which PPy sweeps between reduced and oxidized states through available charge. Therefore, the frequency depends on the supplied current. By biasing the EMPRO in the NDR region with higher currents, the oscillation frequency can be enhanced as PPy can switch from oxidized to reduced state at higher rates. The increased current reduces the duration of steps 1 → 2 and 2 → 3 of the EMPRO oscillation cycle. This, however, decreases the amplitude and the angle of the oscillations. The increase in frequency occurs until a certain bias current after which the PPy supercapacitor requires more time to discharge through the switch. Therefore, the duration of steps 4 → 5 and 5 → 6 increases and the frequency drops. This frequency drop also leads to an increase in the amplitude and angle of the oscillation. This is shown in Fig. 3A where the frequency versus bias current of the EMPROs are plotted. As the EMPROs are parametric relaxation oscillators, once the geometrical parameters are defined precisely through microfabrication techniques and strain engineering, their behavior is expected to be highly reproducible. Nevertheless, the oscillation period of relaxation oscillators is known to be dependent on the capacitance and resistance of the system (*16*), and as they are subject to change depending on variables such as temperature and size (e.g., PPy dimensions), the frequency at which the EMPRO oscillates could vary within a certain range. To measure this change and to test the reproducibility of the generated frequencies, five EMPROs were biased with constant electrical currents from 10 to 700 μA, and the oscillatory voltage responses were recorded via a voltmeter. Afterward, the frequencies were calculated from the oscillatory waveforms by dividing the local maxima amount by the oscillation time. The average and SDs of the frequencies were then calculated and plotted in Fig. 3A with points and error bars, respectively. This figure shows that the EMPROs display consistent characteristics in terms of oscillating frequency in different bias currents.

The frequency reaches a maximum of 95 Hz at the bias current of 300 μA and afterward decreases. The voltage responses of one of the EMPROs to bias currents 30, 100, 300, and 700 μA are shown in fig. S2 (C) to (F). Because of the higher time constant of ion migration compared to electron flow, the mechanical oscillation amplitude is considerably smaller at higher oscillation frequencies. Using the microscope videos, we have estimated the angle at which an EMPRO oscillates at different bias currents. This is illustrated in fig. S2B. Oscillation angles of around 2° to 10° are achieved at biorelevant frequencies. This is in the same ballpark as some insects like the froghopper (*31*). Furthermore, by adding passive appendices to the EMPRO, one could increase the amplitude of the oscillation as shown in movie S1 where the side actuators are equipped with passive long filaments for this purpose.

High-frequency low-amplitude oscillations of the actuators are less clearly observable through regular microscopic observation. To detect EMPRO oscillations better, we observe the motion of the surrounding electrolyte, which is generated by higher-frequency oscillations. The visualization of the fluid dynamics is aided with 10-μm fluorescent particles. These particles were tracked, and their trajectories clearly display the generation of water flow that takes the shape of vortices forming around the beating actuators (Fig. 3B). We hypothesize that the higher supply of charge at higher current biases and the flexibility of the microactuators at 95 Hz produce an undulatory nonreciprocal motion as a flexible oar (*32*) and thereby could produce vortices, proving that the oscillating actuators transfer the energy to the liquid and can produce flow in the low Reynolds number (~10^−2^ according to the geometry of the dynamic contactor) regime. The video of the oscillating EMPRO at 95 Hz in response to a bias current of 300 μA and the resulting flow vorticity can be found in movie S2 (in real-time and slow motion 0.3× playback speed). This increase in frequency could lead to faster swimmers as many studies have shown that the swimmer speed increases with increasing stroke frequencies (*33*–*35*). The velocity of the swimmer is also affected by the Reynolds number, stroke frequency, and the velocity and acceleration of the curvature change. By knowing these parameters, the behavior of the released EMPRO as a swimmer could be simulated in *N*-dimensional parameter space (*33*). The EMPRO that was tested extensively at high currents under turbulent conditions did not show any cracks or fracture. This is mainly due to the robust polymeric reinforcement layers, namely, PI and SU8, which protect the EMPRO shape and structure under turbulent conditions.

Apart from lentic conditions, the behavior of EMPROs was also investigated in laminar flow. The flow was applied via a magnetic stirrer within a large (∅125 mm) petri dish at three different angular velocities of 60, 120, and 240 rpm as shown in fig. S4A. The EMPRO was located 20 mm away from the stirrer providing laminar flow speeds of approximately 0.3, 3.8, and 10.8 $\frac{\text{mm}}{s}$, respectively, against the EMPRO structure. Fluorescent particles of ∅10 μm dispersed in the electrolyte solution demonstrate the flow. First, the redox behavior of PPy was investigated in different flows. No notable change of the cyclic voltammetry diagram was observed in this experiment. The redox diagrams in lentic and flow conditions are shown in fig S4B. Furthermore, the EMPROs were biased with constant electrical currents under the laminar flow conditions to produce oscillations. The results of these experiments show that, with increasing flow, the EMPROs need to be biased at higher constant electrical currents for the oscillations to occur at the oscillation angle φ_𝐶_. Furthermore, the frequency at which the EMPROs oscillate decreases with increasing laminar flow, and they require higher currents to maintain the same frequency as under lentic conditions (fig. S5 and movie S3).

The flow speed around the EMPROs generated by the oscillations under lentic conditions was also studied in vitro and ex vivo using a commercial ultrasound (US) imaging system (FUJIFILM VisualSonics, The Netherlands). US (typically called B-mode US) is a widely used imaging modality in clinical settings that provides high temporal resolution with minimum side effects on tissues. The B-mode US relies on the pulses of acoustic waves emitted by a piezoelectric transducer, and the fast US feedback enables real-time feedback (*36*). However, the low signal-to-noise ratio is an important factor when imaging dynamic small-scale micro-objects. Unlike the B-mode US, Doppler US relies on the Doppler effect, by measuring frequency shifts in reflected US waves after interacting with moving objects. Doppler US does not rely on the amplitude of reflected US waves that make it a suitable tool for imaging dynamic environments. Figure 3C illustrates the experimental setup where the EMPRO is submerged in the electrolyte behind ex vivo chicken breast tissue. A microscope camera image of the EMPRO and its US recreation were recorded in a static state (Fig. 3D). A thin layer of chicken tissue (approximately 6 mm in thickness) was carefully placed and adjusted between the transducer and the EMPROs. The thickness of the chicken tissue was measured with a Vernier caliper. The EMPROs were then biased to oscillate, and the Doppler shifts along with their B-mode US recreations were recorded without (Fig. 3E, top) and with the ex vivo tissue (Fig. 3E, bottom). Corresponding videos can be found in movie S4. The Doppler shifts produced by the EMPROs were quantified by color coding as the color code bar implies the flow measured by the Doppler US mode. In both cases, it is possible to visualize the oscillations of the EMPROs under the B-mode US feedback. There are various parameters that affect the PPy actuation and hence the Doppler shifts caused by the oscillations. These parameters may include the temperature of the tissue or the present ions. Furthermore, the Doppler images are applied to overlay oscillating EMPRO data on the B-mode US. This mode provides rapid identification of the EMPRO movement to distinguish it from the surrounding scattered medium. This feature is beneficial to identify artificial systems in future application scenarios in hard-to-reach sites, e.g., in deep-tissue for real-life microrobotic applications. Another potential application for the EMPROs could be their use as active image labeling using the Doppler effect (*37*), e.g., implants using US-based techniques.

**Fig. 4.**
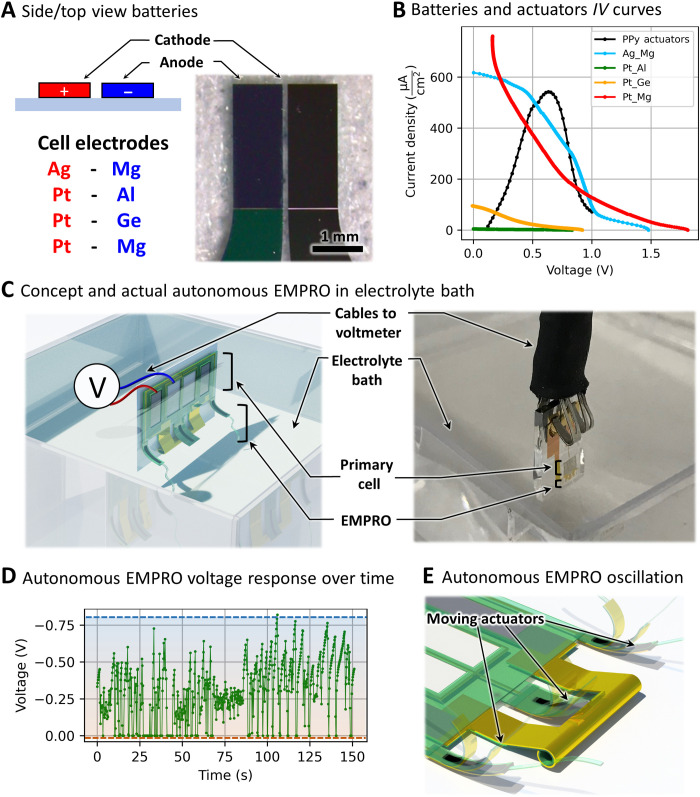
Characterization of battery and autonomous EMPRO. (**A**) Planar electrochemical primary cells. (**B**) *IV* curves of the batteries along with the actuators’ energy consumption normalized by area. (**C**) Concept and realization of the EMPRO integrated with Ag-Mg battery submerged in electrolyte bath with two wires for recording the untethered EMPRO oscillations. (**D**) Autonomous EMPRO voltage oscillations versus time. (**E**) Oscillatory motion and different states of actuators. Photo credit: Farzin Akbar, Institute for Integrative Nanosciences, Leibniz IFW Dresden.

We believe that the EMPRO electromechanical oscillations with frequencies up to 95 Hz achieved in a sub–1-V region (versus Ag/AgCl) can be applied in various biomimetic microrobotic scenarios (*38*). Thus, we integrated EMPROs with a simple energy source on chip to perform untethered oscillations. Low operating voltages of the EMPROs allow application of a single primary cell, avoiding complications (*39*) associated with the operation of numerous battery cells in the same electrolyte. On the basis of the electrical performance of tethered EMPROs, we have chosen four different primary cell configurations (see Table 1), each of which fabricated in a monolithic fashion at wafer scale (Fig. 4A). The open-circuit voltages of these cells are all in the required range of the NDR region of the EMPRO and the redox area of the actuators. The current-voltage characteristics of the cells were compared to that of actuators, and these data are presented in Fig. 4B. The Pt-Mg and the Ag-Mg electrochemical cells both provide the required current to the actuators. However, the Pt-Mg voltage of around 2 V leads to water splitting and therefore harms the PPy actuators. Hence, the Ag-Mg electrochemical cell with an energy density of $0.83\;\frac{\mu \text{Wh}}{{\text{cm}}^{2}}$ was chosen as the suitable energy source for EMPROs. To investigate the reproducibility of the integrated Ag-Mg batteries, three different currents of 0.1, 1, and 2 μA were drained from three different batteries, and the output voltages were measured using a source measurement unit. The currents in this range of several microamperes could move the actuators in oscillatory fashion when the output voltage of the batteries remains in the required NDR bias. The average and SDs of the batteries output voltages were then calculated and plotted versus time in fig. S6, showing that the output power of the batteries is consistent between the devices in these drained currents due to their similar area and hence similar available chemicals for the electrochemical reactions.

**Table 1.** Open-circuit voltages of primary electrochemical cells.

**Electrochemical cell (cathode-anode)**	**Open-circuit voltage (V)**
Pt-Al	0.9
Pt-Ge	0.9
Pt-Mg	1.8
Ag-Mg	1.4

Figure 4C illustrates the configuration (left) and the actual (right) autonomous EMPRO device after the release of the actuators and the self-assembly of the 3D static contactor. Here, the device is mounted on a two-electrode holder and is submerged in the electrolyte. This allows triggering the autonomous oscillation of the EMPRO that is powered by the embedded energy source where no intervention or resupply is necessary for the EMPRO to perform the electromechanical relaxation oscillations for a period of time. The voltage response at the battery-EMPRO interface is recorded by a voltmeter. The autonomous oscillations reach a maximum of 1.5 Hz and are shown in Fig. 4D. The voltage oscillates between two values, resulting into mechanical motion of the actuators as sketched in Fig. 4E. These oscillatory movements of the actuators are recorded under a stereomicroscope, and the video demonstrating this behavior can be found in movie S5. The oscillation continues for at least 5.5 min until the material of the primary cell is consumed (fig. S7). The lower frequency achieved in the autonomous version compared to the externally powered EMPRO is mainly due to the weaker on-board power source. A way to overcome this bottleneck is by, e.g., incorporating flexible rolled-up batteries (*40*, *41*) that decrease the surface area and do not add rigidity to the whole system. Furthermore, their performance is superior in power density, compared to the planar counterparts. This could lead to higher oscillation frequencies in the autonomous EMPROs.

## DISCUSSION

We have demonstrated a shapeable low-power mechatronic oscillator system that can perform electromechanical oscillations with biologically relevant frequencies of up to 95 Hz, which we believe can be beneficial in various biomimetic microrobotic applications. We further achieved an autonomous configuration when integrated with a simple primary energy source. The power consumption of the system for low-frequency (a few hertz) oscillations is approximately ~10 μW, which is comparable to the power demands of insects (*11*) or zooplanktons (*12*). To power the microactuators, a biocompatible electrochemical cell, made of Ag and Mg, was fabricated, and its performance was tested. It was demonstrated that the Ag-Mg cell produces enough power to activate the PPy microactuators and to bias the EMPRO in negative resistance region to cause electromechanical oscillations. The PPy polymers were integrated in a shapeable polymeric platform to develop biocompatible microactuators. The softness, shapeability, and biocompatibility of these microactuators make them ideal for in vivo interactions with soft biological tissues. The EMPRO exploits the mechanical motion of the actuators to create a mechanical switch as a nonlinear element. Whereas the redox PPy-embodied supercapacitor and internal circuit parameters act as the relaxation delay elements of the oscillator, with the parametric geometric design, actuators act as oscillators themselves that leads to a reduced number of functional elements required for autonomous EMPRO realization. Furthermore, it has been shown that the generated oscillating signal can be applied to other microactuators that mimic, e.g., natural flagella undulatory motion. To enhance the lifetime of the EMPRO, it can be powered by energy sources other than primary electrochemical cells such as fuel cells or photocells that provide DC electrical power as long as the fuel or light is present. In addition, the oscillators themselves have been proven to serve as active labels for deep tissue imaging based on the Doppler principle, which makes them intriguing for further uses in vivo for monitoring of medical devices and implants. Last, since the mechanical motion is electrically induced, the microsystem has the potential to be integrated with microelectronics and sensors (*42*, *43*) for future realizations of smart microautonomous systems (*44*) that could swim or walk by the electromechanical relaxation oscillations provided by the EMPROs released from the substrate.

## MATERIALS AND METHODS

### Substrate treatment

Square glasses of 50 mm by 50 mm (D263T eco glass, SCHOTT AG, Mainz, Germany) were used as the substrate in this work. The glasses were first rinsed in a laboratory glassware washer PG85 (Miele & Cie. KG, Gütersloh, Germany) to strip off organic and inorganic contaminants. Next, the surface was treated with oxygen plasma in GIGAbatch 310 (PVA Metrology & Plasma Solutions GmbH, Wettenberg, Germany) for 30 min. Afterward, surface chemical modification with a monolayer of 3-(trimethoxysilyl) propyl methacrylate was done in an oven in vacuum by 150°C for a period of 2 hours.

### Autonomous EMPRO fabrication

The system is integrated with the shapeable polymeric platform whose applicability in designing 3D self-assembled dynamic compact biocompatible devices such as microactuators (*26*) and neural cuff implants (*27*) has been demonstrated. It was fabricated in a fully monolithic wafer scale process (fig. S1). First, a lanthanum-acrylic acid–based organometallic photo-patternable complex is spin-coated at 3000 rpm on the glass substrate for 60 s. Then, the sample is dried at 35°C under a flow of nitrogen for 10 min. It was then exposed through a Cr/glass mask with the use of an MA6 Mask Aligner (SÜSS MicroTec SE, Garching, Germany) with a dosage of 250 mJ/cm^2^ and further developed in water for 35 s. Next, the sample is dried with nitrogen gun and further rinsed in 2-methoxy-1-methylethyl acetate (Micro resist technology GmbH Berlin Gemany). Afterward, it is dried with the nitrogen gun, and last, it is annealed at 220°C for 10 min to form the SL with a thickness of 200 nm (fig. S1.I). Atop the SL, a PI solution is first spin-coated at 6000 rpm for 60 s. Then, the sample is dried at 50°C under constant nitrogen flow for 10 min. Next, similar to the SL, the sample is exposed with a dosage of 500 mJ/cm^2^ with the MA6 mask aligner. It is then developed for 2 min in a developer solution consisting of *N*-ethyl-pyrrolidinone, diethylenglycolmonoethylether (DEGMEE), and ethanol with a volume ratio of 4:2:1, respectively. Afterward, it is rinsed in 2-methoxy-1-methylethyl acetate for 30 s and subsequently dried with a nitrogen gun. Last, it is annealed and imidized at 220°C for 5 min to form the PI layer with a thickness of 800 nm. The PI layer acts as the mechanical support for the static contactor (fig. S1.II). Next, a gold layer is deposited through DC sputtering to form a 50-nm-thick layer. The electrodes are patterned by a liftoff process. For the liftoff process, first, a photoresist layer (AZ5214E Microchemicals GmbH, Ulm, Germany) was spin-coated at 4500 rpm for 35 s and further annealed at 90°C for 5 min. Next, it was exposed by a Maskless Aligner MLA101 (Heidelberg Instruments Mikrotechnik GmbH, Heidelberg, Germany) at a dosage of 20 mJ/cm^2^. Then, the sample underwent a postexposure bake at 120°C for 2 min and was further flood-exposed for 35 s. Afterward, it was developed in a MIF 726 solution (Microchemicals GmbH Ulm Germany) and subsequently rinsed in deionized water to form the sacrificial pattern for the subsequent lift-off process. After the metal electrodes were deposited with the sputtering technique, the AZ photoresist was dissolved in acetone and further rinsed with isopropanol to remove the contaminant particles (fig. S1.III). A HG layer is then photopatterned on top of the PI forming a PI/HG-strained bilayer that causes the tubular geometry of the SC after release. To form the HG, its solution is first spin-coated at 8000 rpm for 60 s. Next, the sample is dried at 40°C under constant nitrogen flow for 10 min. Similar to SL and PI, the sample is exposed with a dosage of 450 mJ/cm^2^ with the MA6 mask aligner and is further developed in DEGMEE for 2 min. Afterward, it is rinsed in 2-methoxy-1-methylethyl acetate for 30 s and subsequently dried with a nitrogen gun. Last, it is annealed at 220°C for 5 min to form the HG layer with a thickness of 400 nm. The two half trapezoid structures of the HG are designed to prevent the tube from rolling from the sides rather than the front (fig. S1.IV). Afterward, a second lanthanum-based SL is patterned on the top to act as a separator between the static contactor and the dynamic contactor (fig. S1.V). Then, a second gold layer is deposited and patterned as the dynamic contactor electrode by the liftoff process (fig. S1.VI). Next, a 200-nm-thick magnesium (Mg) layer is patterned on top of the gold to form the energy source anode using the liftoff process (fig. S1.VII). After that, a 30-nm-thick Hafnium (IV) oxide (HfO_2_) layer is deposited using the atomic layer deposition technique. This layer is then patterned using sulfur hexafluoride plasma dry etching. The HfO_2_ layer protects the Au/Mg interface and prevents chemical shorts (fig. S1.VIII). The 70-nm-thick silver (Ag) cathode of the electrochemical cell is afterward deposited and patterned with the liftoff process (fig. S1.IX). Subsequently, an SU8-2000.5 layer is spin-coated at 3700 rpm for 40 s and is then annealed for 1 min at 95°C. The sample is then exposed with a dosage of 150 mJ/cm^2^ with the MA6 mask aligner and is further annealed for 1 min at 95°C. Afterward, it is developed in the MR-Dev600 developer for 1 min and is then rinsed with isopropanol for 30 s to form a 300-nm-thick layer. This flexible layer acts as the PPy actuator’s mechanical reinforcement layer and as a hard mask for the further deposited PPy layers. Furthermore, the SU8 layer protects the Ag/Au interface to prevent electrochemical short circuits (fig. S1.X). The electrochemical cell was then covered with a 5-μm-thick special resilient ARP5910 (Allresist GmbH Strausberg Germany) photoresist layer to be protected and electrochemically isolated during the deposition of PPy and subsequent release and self-assembly steps. This resist is then flood-exposed under 365-nm ultraviolet light to be developed in a NaOH developer solution after the EMPRO self-assembly (fig. S1.XI). The PPy layer is then electrochemically deposited in a three-electrode fashion as discussed elsewhere (*45*). Briefly, pyrrole monomer (Sigma-Aldrich Co. LLC, Germany) was distilled at vacuum and a temperature of 90°C over a period of 4 hours. The distilled monomer was afterward stored in the freezer at −20° for further use. A solution of 0.1 M sodium dodecylbenzenesulfonate (NaDBS) (Sigma-Aldrich Co. LLC, Germany) and 0.1 M distilled pyrrole was prepared for the electrochemical deposition. A three-electrode Autolab potentiostat (Metrohm AG, Herisau, Switzerland) was used for the electrochemical deposition and characterization of PPy. An Ag/AgCl and a gold electrode were used as pseudoreference and counterelectrode, respectively. A voltage of 0.5 V versus Ag/AgCl was applied for 35 s to form the PPy layers. Afterward, cyclic voltammetry between −1 and 0 V (versus Ag/AgCl) was conducted 10 times to activate the PPy polymer by repeatedly going through the oxidation and reduction states (fig. S1.XII). After the microfabrication, the EMPRO is self-assembled by selectively etching the lanthanum-based SL in an aqueous 1.5% hydrochloric acid solution for 30 min. This leads to the release of the dynamic and static contactors. Because of the swelling of the HG layer upon the release, the static contactor rolls downward to form a tubular structure. On the other hand, since the PPy is in its oxidized and hence contracted state, the dynamic contactor that acts as the microactuator bends upward to form the final self-assembled device. The ARP5910 photoresist plays a major role here as it protects the Ag/Mg electrochemical cell during the lanthanum-based SL wet etch process in the 1.5% hydrochloric acid solution. A precision source/measure unit (B2902A Agilent, California, USA) was used to bias the EMPROs in the negative resistance region for electrical characterization. To investigate the flow generated by the mechanical oscillations of the EMPROs, 10-μm fluorescent particles (Fluoromax, Fisher Scientific GmbH, Schwerte Germany) were added to the 0.1 M NaDBS solution, and the mixture was then shaken to form a uniform dispersion. Afterward, their movements were investigated during EMPRO mechanical oscillations in the videos.

The formation of vortices at high frequencies around the dynamic contactor was visualized by tracking the fluorescent particles in the solution using the Python “trackpy” library. In this program, first, the particles are automatically detected on the basis of their size in the video file. Next, the trajectories are plotted by comparing the location of the detected particles in subsequent frames. Last, the trajectories are plotted on the video as shown in Fig. 3B.

To activate the integrated electrochemical cell for autonomous oscillations, the protective resist is developed and removed in an AR300-26 developer solution. This specific developer was chosen to remove the photoresist as it has minimal harmful effect on the actuators and the integrated electrochemical cell. The autonomous EMPRO was then rinsed with DI water. Afterward, the system was submerged in a 0.1 M NaDBS electrolyte to activate, and the oscillating voltages between the dynamic and static contactor were measured by a precision source/measure unit (B2902A Agilent, California, USA). Furthermore, the mechanical motions were recorded either by a Leica stereoscopic microscope camera (Leica Microsystems GmbH, Wetzlar, Germany) or a phantom high-speed camera (Vision Research Inc., USA).

### US and Doppler shift imaging

The Vevo-LAZR X (FUJIFILM VisualSonics, The Netherlands), a multimodal platform that allows dual US and photoacoustic imaging, was used for the Doppler experiments in in vitro and ex vivo settings. The ex vivo tissue was a chicken breast freshly bought from the supermarket and cut into a 6-mm-thick tissue. The color Doppler measurements were carried out using a 256-element linear array US transducer at a central frequency of 21 MHz, and the signals were collected and reconstructed using on-board automated postprocessing. The images were further optimized by adjusting the parameters (Doppler gain, sensitivity, and persistence). Persistence is adjusted to produce a smooth image. These settings provide better visualization of the moving object by removing any artifact. Color Doppler mode scans an entire imaging volume and converts flow measurements into colors that are superimposed over anatomical US B-mode images, and flow intensities are color-coded. As a result, color Doppler images provide information on both flow intensity and flow direction. The intensity of the color is a function of speed. This experiment allows the detection of the oscillatory motion of the EMPROs in the region of interest, with the color representation of movement direction (away or toward the transducer). The colors indicate the direction of movement relative to the transducer (blue away and red toward the transducer).
