# Supplementary material for: Self-sufficient self-oscillating microsystem driven by low power at low Reynolds numbers
Source: Sci Adv. 2021 Oct 27;7(44):eabj0767. doi: 10.1126/sciadv.abj0767 (PMC8550224; doi:10.1126/sciadv.abj0767)
Supplement: Supplementary file 1 — Figs. S1 to S7 Legends for movies S1 to S5 [file sciadv.abj0767_sm.pdf]

Science Advances
AAAS

Supplementary Materials for

Self-sufficient self-oscillating microsystem driven by low power at low Reynolds numbers

Farzin Akbar, Boris Rivkin, Azaam Aziz, Christian Becker, Dmitriy D. Karnaushenko, Mariana Medina-Sánchez*, Daniil Karnaushenko*, Oliver G. Schmidt*

*Corresponding author. Email: m.medina.sanchez@ifw-dresden.de (M.M.-S.); d.karnaushenko@ifw-dresden.de (D.K.); oliver.schmidt@main.tu-chemnitz.de (O.G.S.)

Published 27 October 2021, *Sci. Adv.* **7**, eabj0767 (2021)
DOI: 10.1126/sciadv.abj0767

The PDF file includes:

Figs. S1 to S7
Legends for movies S1 to S5

Other Supplementary Material for this manuscript includes the following:

Movies S1 to S5

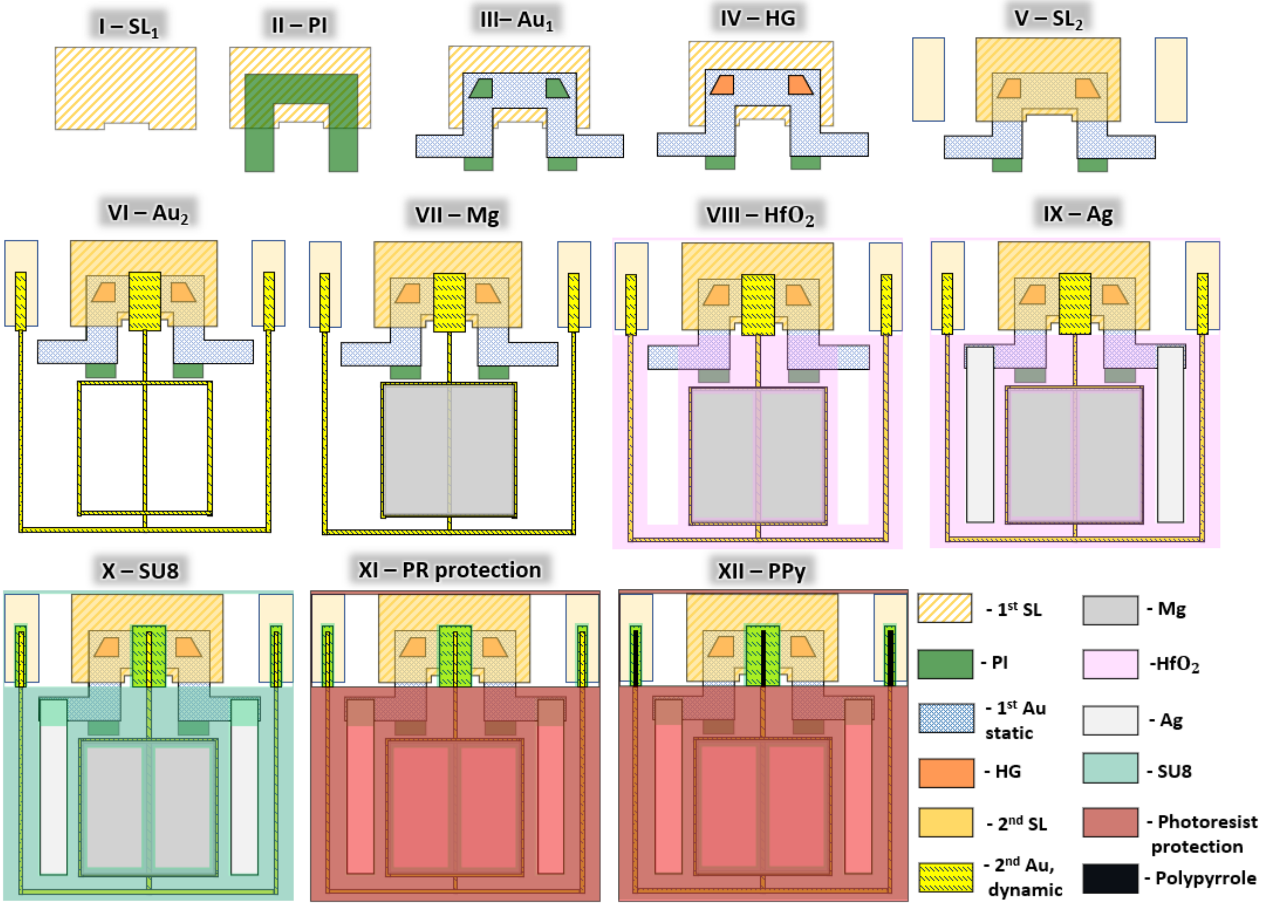

Supplementary Fig. S1 Fabrication steps of the autonomous EMPRO: **I** Sacrificial layer **II** Polyimide substrate **III** Gold as the static contactor electrode **IV** Hydrogel as the swelling layer **V** Second sacrificial layer. **VI** Second gold as the dynamic contactor electrode. **VII** Mg as the energy source anode. **VIII** HfO_2 as the insulating layer. **IX** Ag as the energy source cathode **X** SU8 as the mechanical reinforcement layer, and as hard mask for PPy patterning **XI** ARP5910 protective photoresist layer for the battery **XII** PPy as the electroactive polymer.

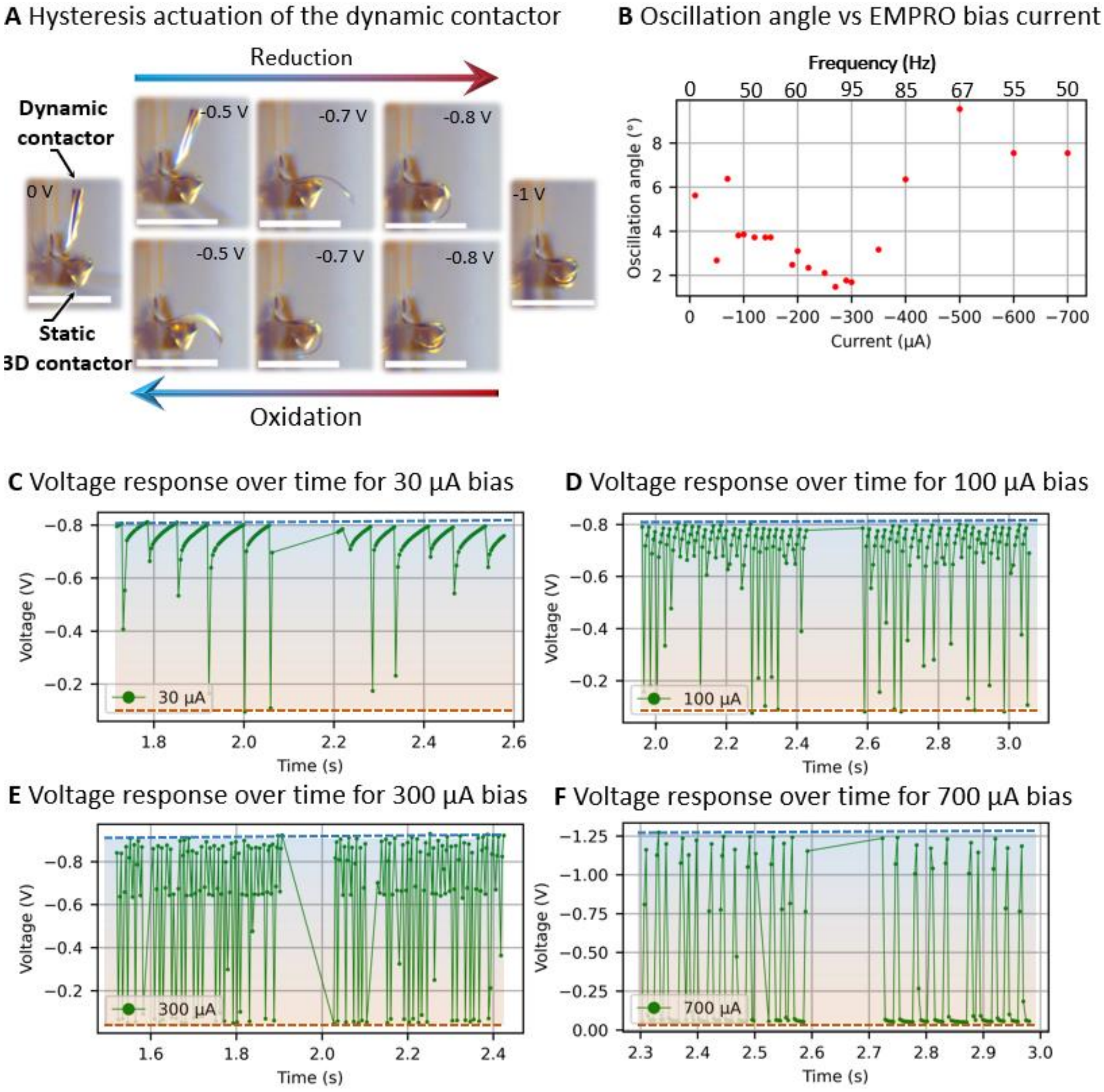

Supplementary Fig. S2 EMPRO hysteresis movement, oscillation angles, and voltage response in lentic conditions. (A) Dynamic contactor hysteresis movement following PPy redox with a 50° angle change in reduction from -0.5V to -0.7V vs Ag/AgCl Scale bar: 500 µm **(B)** Oscillation angle versus bias current of an EMPRO. EMPRO voltage response to bias of **(C)** 30 µA, **(D)** 100µA, **(E)** 300µA, and **(F)** 700 µA. Photo credit: Farzin Akbar, Institute for Integrative Nanosciences, Leibniz IFW Dresden.

A 24 EMPROs after self-assembly **B** EMPROs after self-assembly

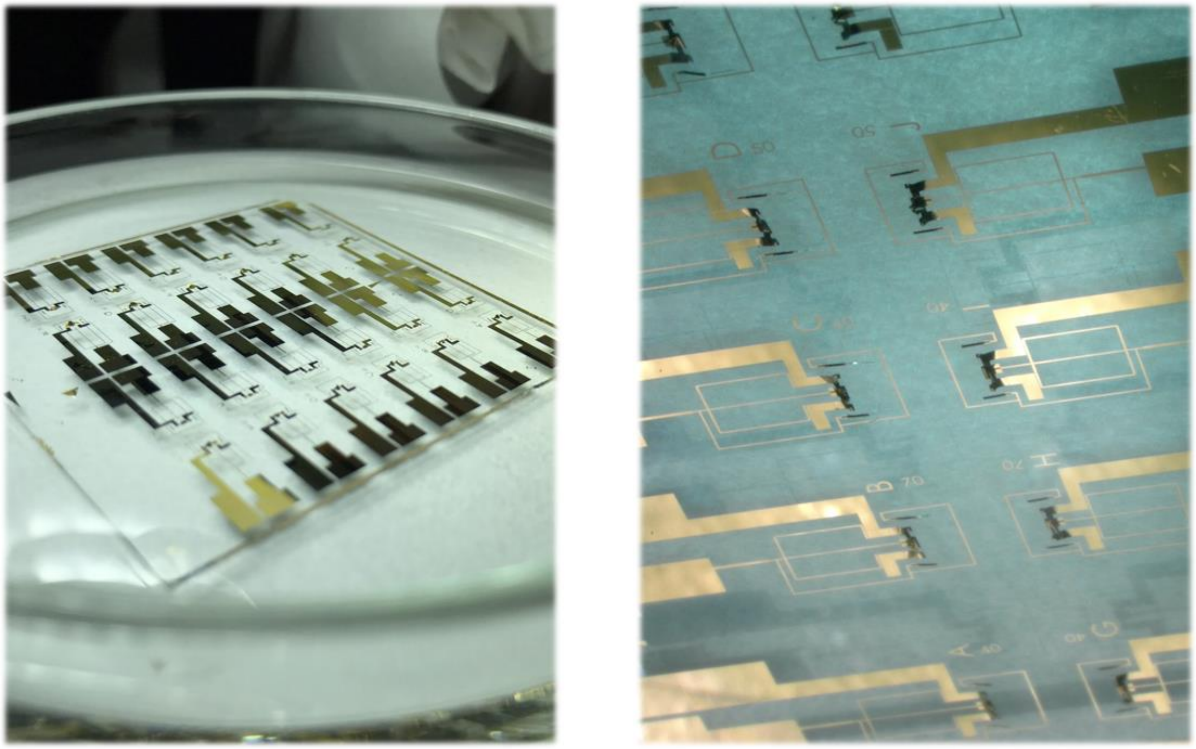

Supplementary Fig. S3. Wafer scale production of self-assembled EMPROs. (A) Overview of 24 self-assembled EMPROs on a 50×50 mm^2 glass wafer **(B)** Overview of 10 self-assembled EMPROs. Photo credit: Farzin Akbar, Institute for Integrative Nanosciences, Leibniz IFW Dresden.

A Flow experiment setup **B** PPy redox under lentic and flow conditions

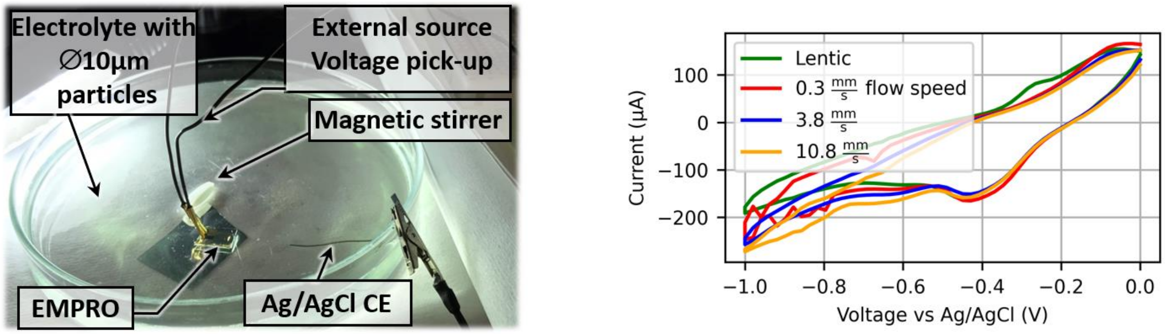

Supplementary Fig. S4. EMPRO laminar flow experiments (A) Experiment setup **(B)** PPy redox behavior under lentic and laminar flow conditions. Photo credit: Farzin Akbar, Institute for Integrative Nanosciences, Leibniz IFW Dresden.

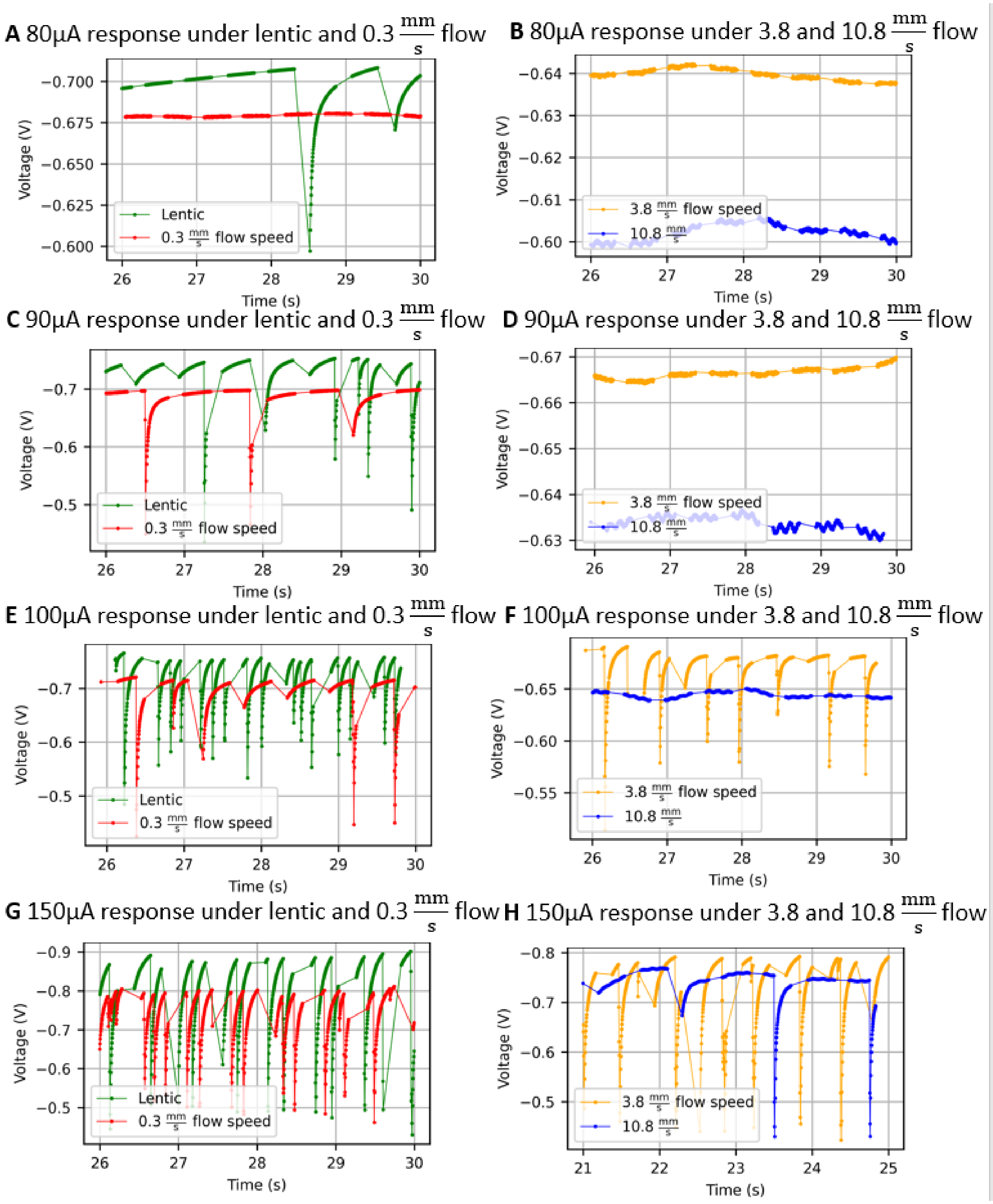

Supplementary Fig. S5. EMPRO voltage response under lentic and laminar flow conditions. (A) comparison of lentic and 0.3 $\frac{mm}{s}$ flow speed at 80 μA bias **(B)** comparison of 3.8 and 10.8 $\frac{mm}{s}$ flow speed at 80 μA bias **(C)** comparison of lentic and 0.3 $\frac{mm}{s}$ flow speed at 90 μA bias **(D)** comparison of 3.8 and 10.8 $\frac{mm}{s}$ flow speed at 90 μA bias **(E)** comparison of lentic and 0.3 $\frac{mm}{s}$ flow speed at 100 μA bias **(F)** comparison of 3.8 and 10.8 $\frac{mm}{s}$ flow speed at 100 μA bias **(G)** comparison of lentic and 0.3 $\frac{mm}{s}$ flow speed at 150 μA bias **(H)** comparison of 3.8 and 10.8 $\frac{mm}{s}$ flow speed at 150 μA bias.

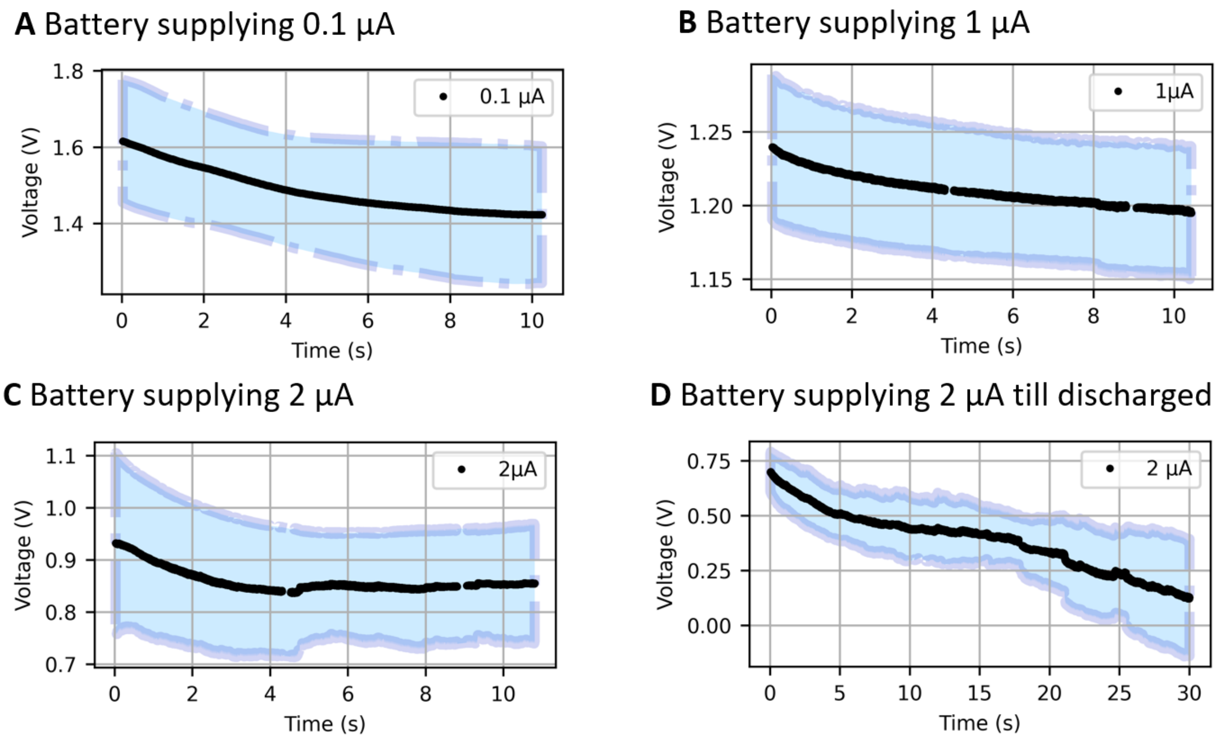

Supplementary Fig. S6. Output voltage average and standard deviation of three integrated batteries. Supplying **(A)** 0.1 µA **(B)** 1 µA **(C)** 2 µA **(D)** 2 µA at complete discharge.

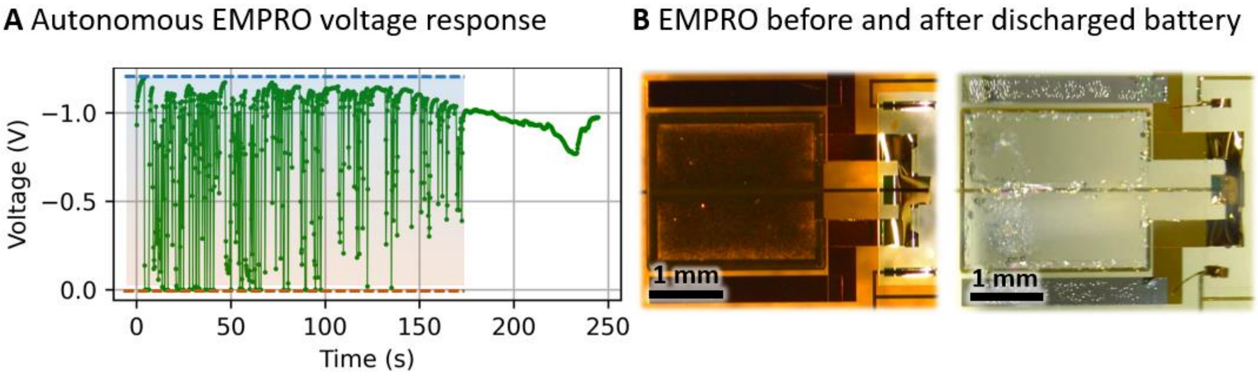

Supplementary Fig. S7 Final stage of autonomous EMPRO (A) Autonomous EMPRO voltage response until consumed battery **(B)** A top view of the autonomous EMPRO with full battery under protective resist (left) and fully discharged battery (right). Photo credit: Farzin Akbar, Institute for Integrative Nanosciences, Leibniz IFW Dresden.

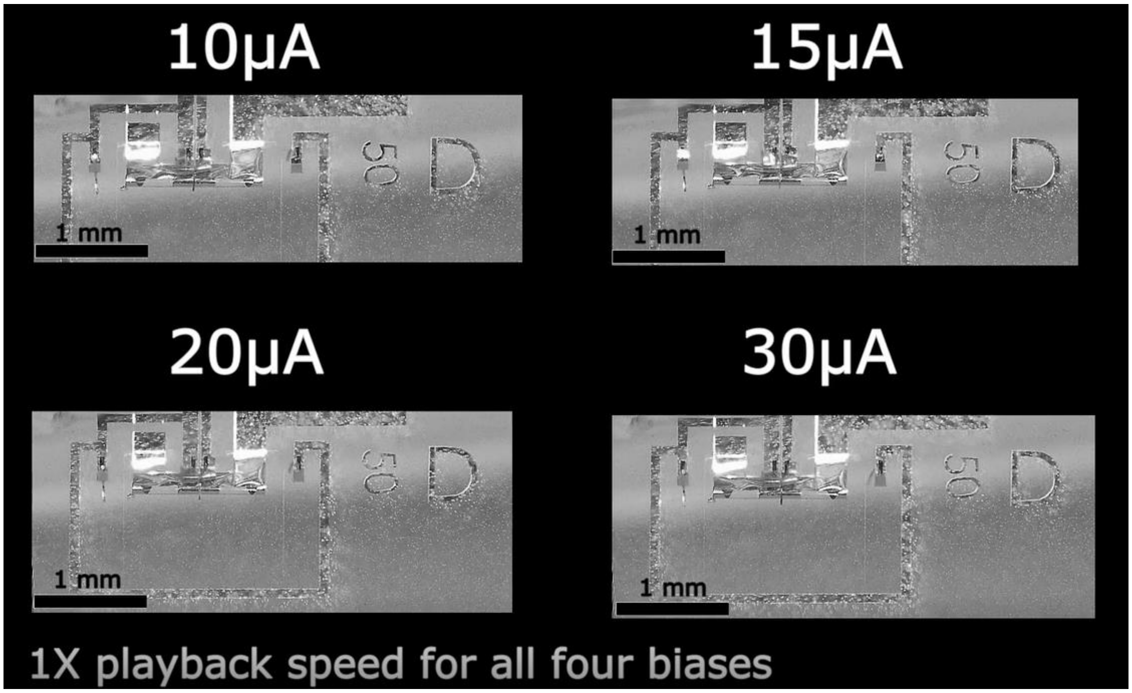

Legend for Supplementary Movie S1.
EMPRO oscillations with four different constant bias currents where the EMPROs are submerged in the electrolyte containing 10 μm particles.

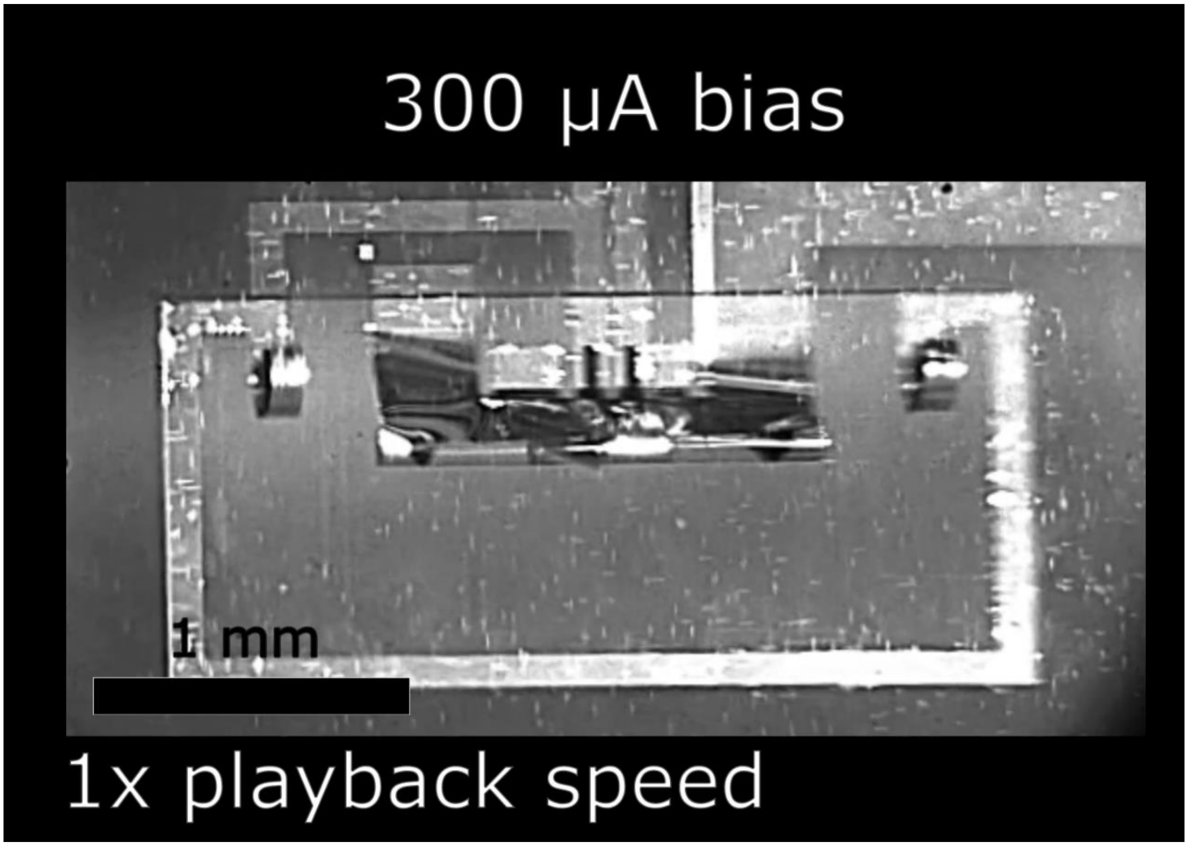

Legend for Supplementary Movie S2.
EMPRO oscillations with constant bias current of 300 μA and formation of vortices as shown in the movement of 10 μm particles.

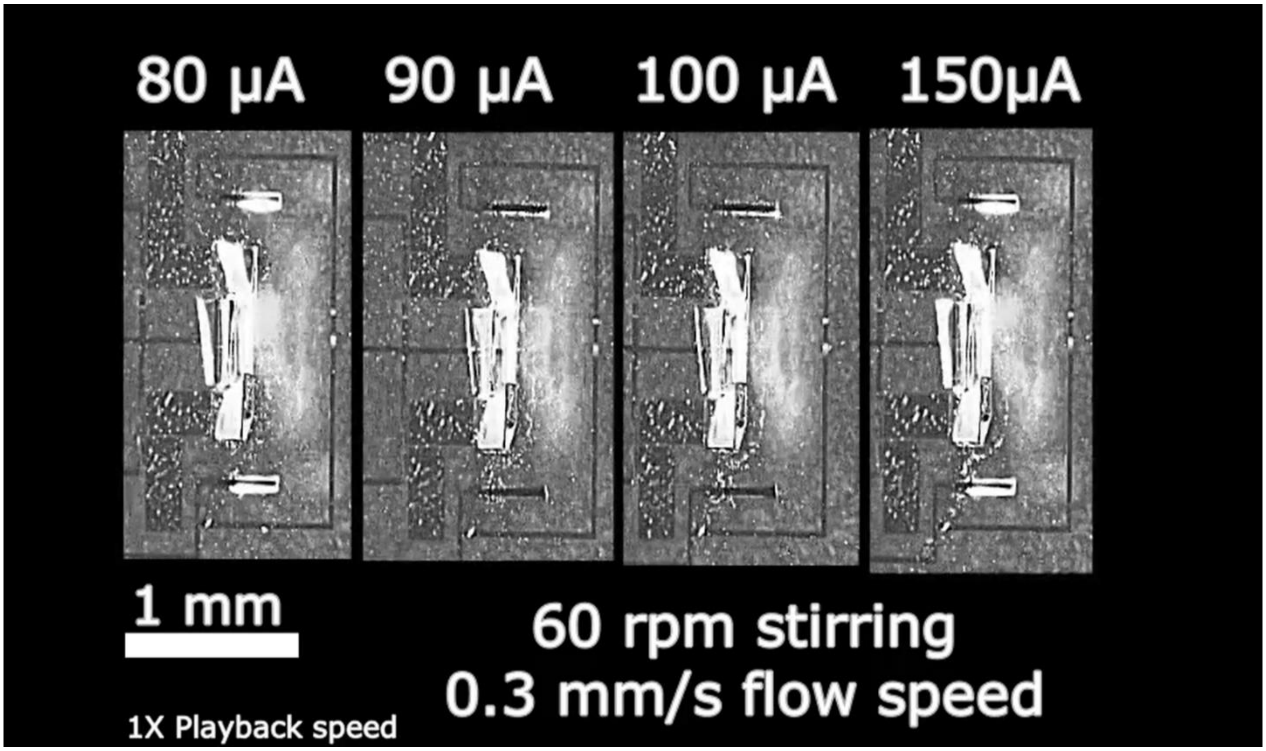

Legend for Supplementary Movie S3.
EMPRO oscillations with constant biases in laminar flow conditions. The flow is demonstrated by adding 10 µm fluorescent particles in the solution.

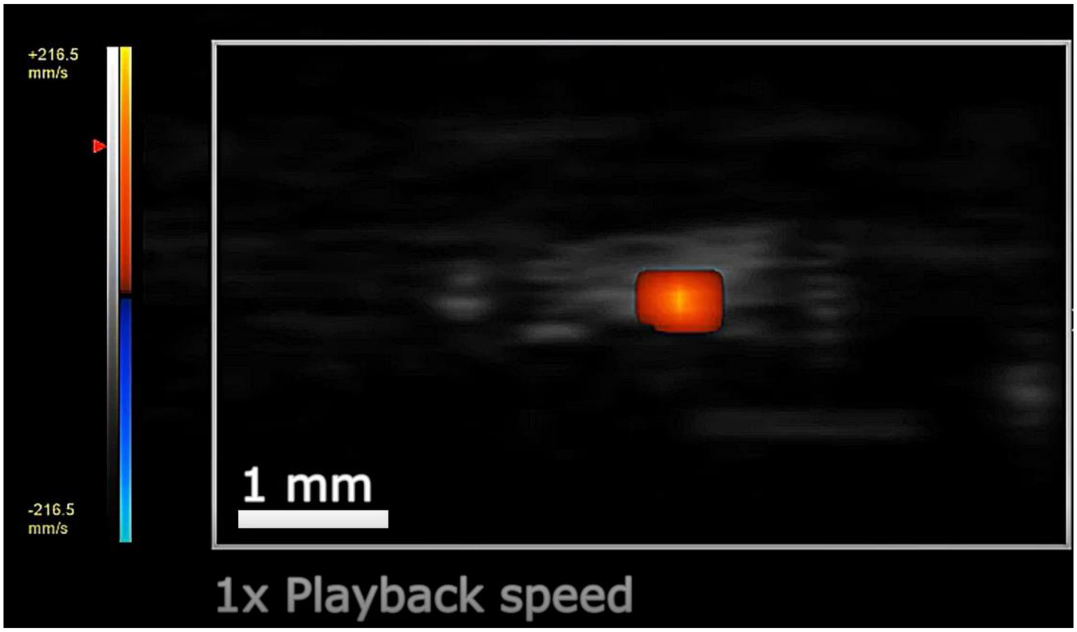

Legend for Supplementary Movie S4.
Doppler shift measurement due to the flow generated by EMPRO oscillations *in-vitro* and *ex-vivo*.

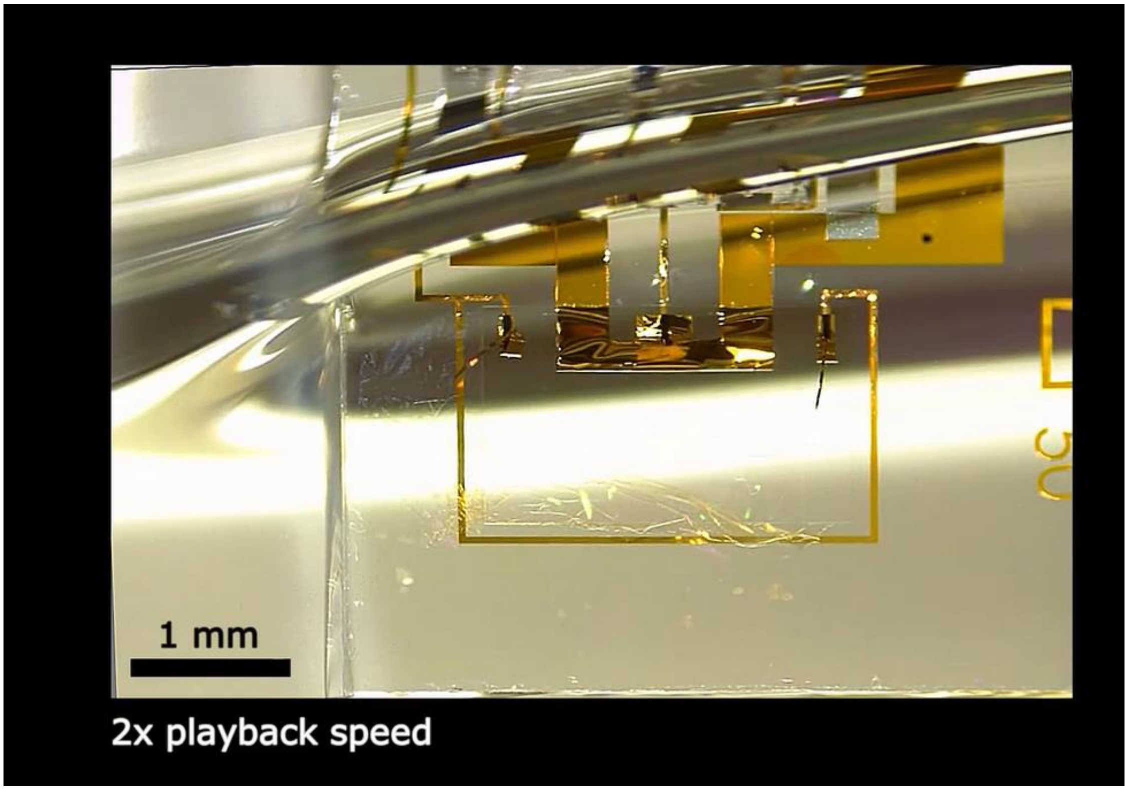

Legend for Supplementary Movie S5.
Autonomous EMPRO oscillations where the EMPROs are biased by the embedded Ag-Mg battery in the NDR region for oscillations.
